# Spin dynamics and relaxation in graphene dictated by electron-hole puddles

**DOI:** 10.1038/srep21046

**Published:** 2016-02-15

**Authors:** Dinh Van Tuan, Frank Ortmann, Aron W. Cummings, David Soriano, Stephan Roche

**Affiliations:** 1 Catalan Institute of Nanoscience and Nanotechnology (ICN2), CSIC and The Barcelona Institute of Science and Technology, Campus UAB, Bellaterra, 08193 Barcelona, Spain; 2 Institute for Materials Science, Dresden Center for Computational Materials Science, Technische Universität Dresden, 01062 Dresden, Germany; 3 ICREA, Institució Catalana de Recerca i Estudis Avançats, 08070 Barcelona, Spain

## Abstract

The understanding of spin dynamics and relaxation mechanisms in clean graphene, and the upper time and length scales on which spin devices can operate, are prerequisites to realizing graphene-based spintronic technologies. Here we theoretically reveal the nature of fundamental spin relaxation mechanisms in clean graphene on different substrates with Rashba spin-orbit fields as low as a few tens of *μ*eV. Spin lifetimes ranging from 50 picoseconds up to several nanoseconds are found to be dictated by substrate-induced electron-hole characteristics. A crossover in the spin relaxation mechanism from a Dyakonov-Perel type for SiO_2_ substrates to a broadening-induced dephasing for hBN substrates is described. The energy dependence of spin lifetimes, their ratio for spins pointing out-of-plane and in-plane, and the scaling with disorder provide a global picture about spin dynamics and relaxation in ultraclean graphene in the presence of electron-hole puddles.

The tantalizing prospect of graphene spintronics was initiated by Tombros and coworkers^1^, who first reported long spin diffusion length in large area graphene. The small spin-orbit coupling (SOC) in carbon, plus the absence of a hyperfine interaction, suggested unprecedented spin lifetimes (*τ*_*s*_) at room temperature (from *μ*s to ms)^2^,^3^,^4^,^5^,^6^,^7^.

However, despite significant progress in improving graphene quality, resolving contact issues, and reducing substrate effects^1^,^8^,^9^,^10^,^11^,^12^,^13^,^14^,^15^, the measured *τ*_*s*_ are orders of magnitude shorter, even for high-mobility samples. Extrinsic sources of SOC, including adatoms^16^,^17^,^18^,^19^ or lattice deformations^20^,^21^, have been proposed to explain this discrepancy. Moreover, the nature of the dominant spin relaxation mechanism in graphene is elusive and debated. The conventional Dyakonov-Perel (DP)^22^ and Elliot-Yafet (EY)^23^ mechanisms, usually describing semiconductors and disordered metals, remain inconclusive in graphene because neither effect can convincingly reproduce the observed scaling between *τ*_*s*_ and the momentum relaxation time *τ*_*p*_^8^,^11^. Although generalizations of both mechanisms have been proposed, they do not allow an unambiguous interpretation of experiments^6^,^20^,^21^,^24^,^25^.

It should be noted that the achieved room-temperature spin lifetime in graphene is already long enough for the exploration of spin-dependent phenomena such as the spin Hall effect^26^,^27^,^28^, or to harness proximity effects as induced for instance by magnetic oxides^29^ or semiconducting tungsten disulphide^30^. However, a comprehensive picture of the spin dynamics of massless Dirac fermions in the presence of weak spin-orbit coupling fields is of paramount importance for further exploitation and manipulation of the spin, pseudospin and valley degrees of freedom^7^,^31^,^32^,^33^.

In this study, we show numerically that a weak uniform Rashba SOC (tens of *μ*eV), induced by an electric field or the substrate, yields spin lifetimes from 50 ps up to several nanoseconds. The dominant spin relaxation mechanism is shown to be dictated by long range potential fluctuations (electron-hole puddles)^34^. For graphene on a SiO_2_ substrate, such disorder is strong enough to interrupt the spin precession driven by the uniform Rashba field, resulting in motional narrowing and the DP mechanism. We also find the ratio 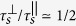, demonstrating the anisotropy of the in-plane Rashba SOC field. For the case of a hexagonal boron nitride (hBN) substrate, the role of electron-hole puddles is reduced to an effective energy broadening and the spin lifetime is limited by pure dephasing^35^,^36^. These situations, however, share a common fingerprint - an M-shape energy dependence of *τ*_*s*_ that is minimal at the Dirac point. Taken together, our results provide deeper insight into the fundamentals of spin lifetimes in graphene dominated by electron-hole puddles.

## Results

### Disorder and Transport time

**Figure 1:**
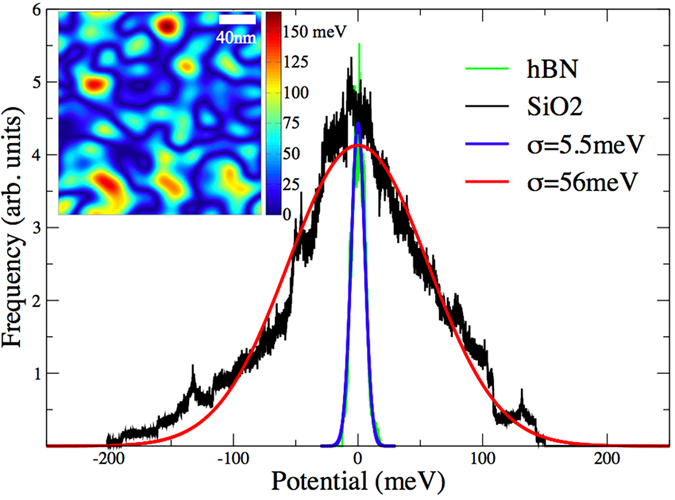
Onsite energy distribution of the carbon atoms in the graphene sample, which mimics the chemical potential induced by hBN (green) and SiO_2_ (black) substrates together with their Gaussian fitting lines. Inset: Real space vizualization of the energy landscape for a graphene sample with 0.04% Gaussian impurities (SiO_2_ case). Absolute values are pictured.

Electron-hole puddles are real-space fluctuations of the chemical potential, induced by the underlying substrate, which locally shift the Dirac point^37^,^38^,^39^. Since measured transport properties usually result from an average around the charge neutrality point, it is generally difficult to access the physics at the Dirac point. As shown by Adam and coworkers^37^, electron-hole puddles can be modeled as a random distribution of long range scatterers, 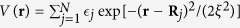, where *ξ* = 10 and 30 nm denote the effective puddle ranges for SiO_2_ and hBN substrates, respectively^38^,^40^, and 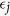 is randomly chosen within [−Δ, Δ]. Based on experimental data, typical impurity densities are *n*_*i*_ = 10^12^ cm^−2^ (*N*_*i*_/*N*_tot_ = 0.04%, the percentage of impurity sites) for SiO_2_ and *n*_*i*_ = 10^11^ cm^−2^ 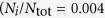
%) for hBN substrates^38^,^41^. In addition, the onsite energy profiles were found to obey a Gaussian distribution, with standard deviations of *σ* = 5.5 and 56 meV for hBN and SiO_2_ substrates, respectively. From such information, we can tune Δ to obtain suitable disorder profiles for the onsite energy of the *π*-orbital. Figure 1 (main frame) shows the calculated onsite energy distribution corresponding to hBN and SiO_2_ substrates, where we set Δ = 50 meV for SiO_2_ and Δ = 5 meV for hBN in order to match the experimental onsite energy profiles. The inset of Fig. 1 illustrates an energy landscape for a sample with 0.04% Gaussian impurities (SiO_2_ case).

**Figure 2:**
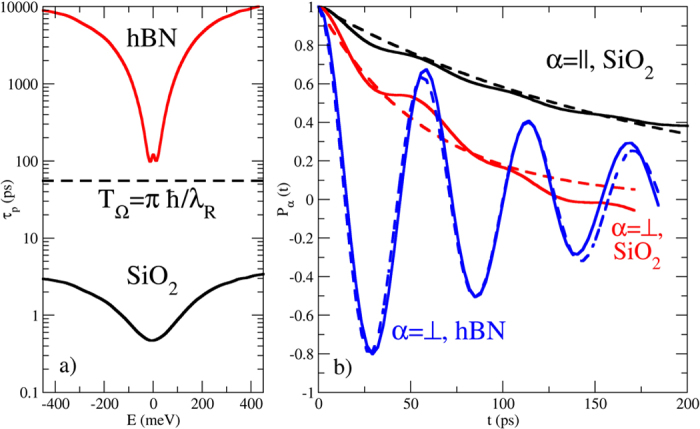
(**a**) Transport times for graphene on SiO_2_ and hBN substrates (solid black and red curves, respectively). The dashed line shows the spin precession time. (**b**) Time-dependent spin polarization for out-of-plane (solid red line) and in-plane (solid black line) spin injection for the SiO_2_ substrate, plus the fits to the exponential damping (dashed lines). The blue curves show the same information for the hBN substrate with out-of-plane injection.

To fully characterize the role of electron-hole puddles, we evaluate the transport time *τ*_*p*_ using a real-space order-N approach, which computes the diffusion coefficient *D*(*E*, *t*). We extract *τ*_*p*_ from the saturation of *D*(*E*, *t*) since 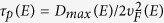
42. For numerical convenience, the calculations are first made using a larger value Δ = 0.27 eV (for which intervalley scattering remains moderate^43^), and from this we obtain *τ*_*p*_(*E*) for hBN and SiO_2_ substrates using the scaling law^37^.


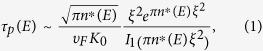


where *I*_1_(*x*) is the modified Bessel function of the first kind, 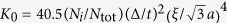 is a dimensionless parameter dictating the strength of the Gaussian potential, and the carrier density n*(*E*) is modified from the pristine graphene density *n*(*E*) by 
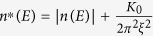
37,44,45. The computed *τ*_*p*_ are shown in Fig. 2(a) for both substrates. For SiO_2_, *τ*_*p*_ is on the order of a few ps, while for hBN *τ*_*p*_ is more than two orders of magnitude larger. The spin precession time used in our calculations, 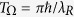, is shown for comparison.

We observe that the obtained values are consistent with experimental estimates. Monteverde and coworkers found a similar energy dependence (as in our Fig. 2(a)) of room temperature transport times for monolayer graphene on silicon oxide^46^. Their experimental data range from 50 fs to 100 fs, whereas our numerical results predict values close to Dirac point of about 400 fs. This difference is likely due to temperature effects, additional adsorbed impurities or other structural defects which are not considered in our simulations. Similarly, the values we obtained for the case of hBN substrates are consistent with current best measurements of hBN-encapsulated graphene, which report long mean free paths up to 30 *μm* and mobilities up to 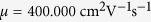 47. Our numerical results for the transport time in graphene on hBN are close to 100 ps at the Dirac point (which gives 100 microns for the mean free path), and therefore differ by less than one order of magnitude with respect to the most recent experimental data.

### Spin dynamics and lifetimes in the presence of electron-hole puddles

We now analyze the spin dynamics for puddles corresponding to the SiO_2_ and hBN substrates. The blue curve in Fig. 2(b) shows the time-dependent spin polarization for the hBN substrate 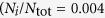 at the Dirac point for an initial out-of-plane polarization, 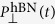 (see Methods). The polarization exhibits oscillations with period 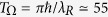 ps, corresponding to the spin precession induced by the Rashba field. Simultaneously, the polarization decays in time, and by fitting 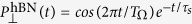, both *T*_Ω_ and the spin relaxation time *τ*_*s*_ can be evaluated.

Figure 2(b) also shows 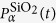 for the SiO_2_ substrate 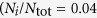 with initial spin polarization in-plane (*α* = ||) and out-of-plane (*α* = ⊥). In contrast to the hBN case, for which 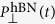 exhibits significant precession, the disorder strength of electron-hole puddles for SiO_2_ is sufficient to interrupt spin precession. As a result, the polarization for SiO_2_ is better fit with 
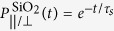
. The absence of precession for 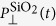 compared to 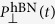 is consistent with the ratio between transport time and precession frequency, since 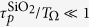 whereas 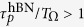.

**Figure 3:**
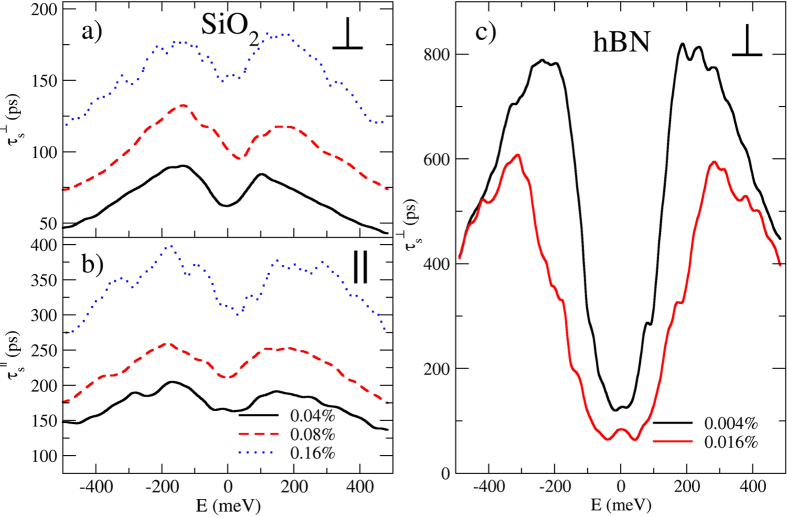
Spin lifetimes for out-of-plane (**a**) and in-plane (**b**) spin injection for SiO_2_ substrate at impurity densities of 0.04% (black solid curves), 0.08% (red dashed curves), and 0.16% (blue dotted curves). (**c**) Spin lifetime with out-of-plane spin injection for the hBN substrate at impurity densities of 0.004% (black curve) and 0.016% (red curve).

To scrutinize the origin of the dominant relaxation mechanism, we first examine the spin lifetimes *τ*_*s*_ for the SiO_2_ case when rotating the initial spin polarization (out-of-plane vs. in-plane), and when varying the impurity concentration (0.04%, 0.08%, and 0.16%). Figure 3 shows the extracted *τ*_*s*_ for the out-of-plane (a) and in-plane (b) cases. The energy dependence of *τ*_*s*_ exhibits an M-shape increasing from a minimum at the Dirac point, with a saturation and downturn of *τ*_*s*_ for *E* ≥ 200 meV. The values of *τ*_*s*_ range from 50 to 400 ps depending on the initial polarization and impurity density. We observe an increase of *τ*_*s*_ with *n*_*i*_, which shows that a larger scattering strength reduces spin precession and dephasing, resulting in a longer spin lifetime, as described by the so-called motional narrowing effect^48^. Additionally, the ratio 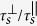 (not shown) changes from 0.3 to 0.45 when 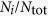 is varied from 0.04 to 0.16%. Such behavior is expected when enhanced scattering drives more randomization of the direction of the Rashba SOC field, which ultimately yields 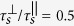 in the strong disorder limit^2^,^3^. These results are fully consistent with the DP spin relaxation mechanism^20^,^21^,^48^.

Figure 3(c) shows 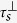 for the hBN substrate (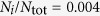 and 0.016%) where a similar M-shape is observed. While 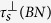 is similar to 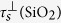 near the Dirac point, it is much larger at higher energies, reaching nearly 1 ns (for λ_*R*_ = 37.4 *μ*eV). A striking difference is that the scaling of *τ*_*s*_ with *n*_*i*_ is opposite to that of the SiO_2_ case, with an increase in puddle density resulting in a decrease in *τ*_*s*_, which indicates a different physical origin. For hBN, this behavior is reminiscent of the EY mechanism, but we will argue below that its origin is different.

### Crossover in spin relaxation behavior for hBN and SiO_2_ substrates

**Figure 4:**
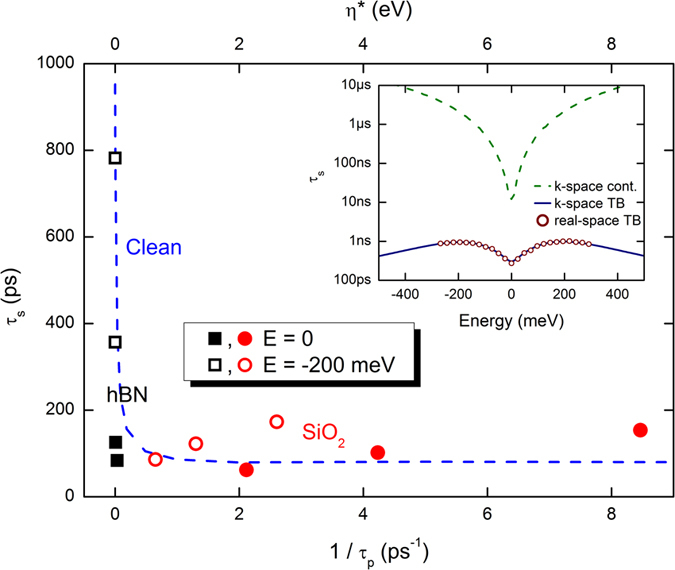
Low-energy spin lifetimes versus 1/*τ*_*p*_ (for initial out-of-plane spin polarization). Squares (circles) are for graphene on hBN (SiO_2_) substrate. Closed (open) symbols are for spin relaxation at the Dirac point (at *E* = −200 meV). The blue dashed line shows the spin lifetime assuming only energy broadening (top axis). Inset: spin lifetime in absence of puddles computed using the TB model in real space (red circles) or *k*-space (blue solid line), and the low-energy model in *k*-space (green dashed line), with *η* = 13.5 meV.

Figure 4 provides a global view of our results, where we plot *τ*_*s*_ vs. 1/*τ*_*p*_ for the SiO_2_ and hBN substrates (black and red symbols respectively) at the Dirac point and at *E* = −200 meV (closed and open symbols respectively). For low defect densities (hBN substrate), *τ*_*s*_ decreases strongly with decreasing *τ*_*p*_. However, with increasing defect density (SiO_2_ substrate) this trend reverses and *τ*_*s*_ scales almost linearly with 1/*τ*_*p*_, according to the DP relationship 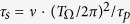. At *E* = −200 meV, *ν* = 1, fitting the usual DP theory. At the Dirac point, the scaling is somewhat weaker, with *ν* = 1/4. These results are reminiscent of those summarized in Fig. 5(a) of Drogeler *et al.*^13^, where spin lifetimes of graphene devices on SiO_2_ scaled inversely with the mobility, while devices on hBN appear to show the opposite trend.

While the SiO_2_ results of Fig. 4 show DP behavior, the nature of the spin relaxation for weak electron-hole puddles is less clear. The fact that *τ*_*s*_ and *τ*_*p*_ decrease together suggests the EY mechanism, but we find *τ*_*s*_ ≤ *τ*_*p*_ near the Dirac point and *τ*_*s*_ ≪ *τ*_*p*_ at higher energies. This contrasts with the usual picture of EY relaxation, where charge carriers flip their spin when scattering off impurities, giving *τ*_*s*_ = *τ*_*p*_/*α*, where *α* ≪ 1 is the spin flip probability^6^. Instead, this situation matches that described in ref. 48; when *τ*_*p*_ > *T*_Ω_, the spin precesses freely until phase information is lost during a collision, in analogy to the collisional broadening of optical spectroscopy. More collisions result in a greater loss of phase, reducing *τ*_*s*_ with decreasing *τ*_*p*_. We verify this by removing the real-space disorder (setting Δ = 0) and modeling the electron-hole puddles with an effective Lorentzian energy broadening η^*^. The results are shown in Fig. 4 (main frame, blue dashed line), where we plot *τ*_*s*_ vs. η^*^ at *E* = −200 meV (top axis). For small η^*^, the scaling matches well with the real-space simulations of hBN, indicating that the puddles can be represented as a uniform energy broadening (see supplementary material). Larger values of η^*^ lead to stronger mixing of different spin dynamics and *τ*_*s*_ saturates at very large η^*^. There, the scaling of *τ*_*s*_ vs. η^*^ clearly fails to replicate the DP behavior seen in the real-space simulations, since the effective broadening model does not induce the momentum scattering necessary for motional narrowing^48^.

Next we explain the origin of the M-shaped energy dependence of *τ*_*s*_. At low energies, the spin dynamics are dominated by strong spin-pseudospin coupling^36^, which yields fast dephasing and a minimum of *τ*_*s*_ at the Dirac point, in agreement with experimental data. At higher energies, the origin of the downturn of *τ*_*s*_ depends on the substrate. For the case of SiO_2_ substrate it is driven by the conventional DP mechanism, where 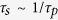. For the case of hBN, the downturn of *τ*_*s*_ can be explained by comparing the spin dynamics in the TB model (Eq. (2) in Methods) with the low-energy model in the absence of puddles (Δ = 0). In this regime 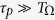, and spin dephasing and relaxation are driven by a combination of energy broadening and a nonuniform spin precession frequency. For the TB model, spin dynamics are calculated with the real-space approach and with a standard *k*-space approach and give identical *τ*_*s*_ (inset of Fig. 4, red circles and blue solid line), indicating the equivalence of the real- and *k*-space approaches in the clean limit when accounting for the full TB Hamiltonian. We observe that while for all models, the spin lifetime shows a minimum at the Dirac point, spin transport simulations with the widely used low-energy Hamiltonian 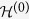 (see Methods for 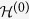 and green dashed line in Fig. 4 inset for results) clearly cannot capture the saturation and downturn of *τ*_*s*_(*E*), i.e. its full M-shape. To qualitatively reproduce the M-shape of *τ*_*s*_(*E*), the first-order term of the Rashba Hamiltonian, 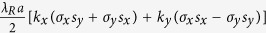, needs to be included in 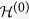. This term introduces stronger dephasing at higher energy, driven by the anisotropy of the Rashba spin-orbit interaction^36^.

In addition to their different energy dependence, the TB and low-energy models also yield very different spin lifetimes. A value of *τ*_*s*_ = 10 ns is obtained at the Dirac point for the low-energy model, which is two orders of magnitude larger than *τ*_*s*_ from the TB Hamiltonian, indicating a strong spin dephasing induced by the high-order *k*-terms. Interestingly, by studying the changes of *τ*_*s*_(*E*) with respect to the Rashba SOC strength, we observe the scaling behavior 
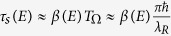
, meaning the spin relaxes after a finite number of precession periods *β* (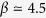 close to the Dirac point), see Supplementary Material. This suggests that dephasing is the limiting factor of spin lifetimes in the ultraclean case. We finally note that by taking *λ*_*R*_ = 5 *μ*eV (electric field of 1 V/nm^4^), a spin lifetime of 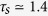 ns is deduced at the Dirac point, whereas at higher energies *τ*_*s*_ could reach about 10 ns.

## Discussion

Our results show a clear transition between two different regimes of spin relaxation, mediated solely by the scattering strength of the electron-hole puddles. For hBN substrates, spin relaxation is dominated by dephasing arising from an effective energy broadening induced by the puddles, and *τ*_*s*_ scales with *τ*_*p*_. In contrast, for SiO_2_ substrates dephasing is limited by motional narrowing, leading to a DP regime with 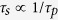. Remarkably, both regimes exhibit similar values of *τ*_*s*_ at the Dirac point and a similar M-shape energy dependence (Fig. 3), making it a signature of spin relaxation in graphene for all puddle strengths. The crossover between both mechanisms occurs when 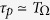, which might have been realized in some experiments. This could explain some conflicting interpretations of experimental data in terms of either Elliot-Yafet or Dyakonov-Perel mechanisms^11^.

We note the large discrepancy between our conclusions and the former theoretical work by C. Ertler *et al.*^3^. Indeed, the conclusions of ref. 3 (the spin lifetime maximum at the Dirac point and reaching values in the millisecond range) are fully inconsistent with the main experimental features, which are a minimum of the spin lifetime at the Dirac point and an increase for higher energy, and with spin lifetimes on the order of hundreds of ps to a few nanoseconds. The fundamental difference of the model used in C. Ertler *et al.*^3^ and our present study turns out to be essential. In their study, the spin precession frequency was assumed to be uniform in energy, while our approach is a fully quantum study of spin dynamics without any approximation. As a result, from our analysis of the time-dependence of the spin polarization we observe that the spin precession frequency is non-uniform in energy, which is one essential aspect explaining a faster decay of spin lifetime close to the Dirac point.

Our findings suggest alternative options for determining the spin relaxation mechanism in graphene from experimental measurements. Indeed, the typical approach, to examine how *τ*_*p*_ and *τ*_*s*_ scale with electron density and to assign either the EY or DP mechanism accordingly, is not always appropriate. For example, the EY mechanism in graphene is given by 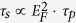, such that *τ*_*s*_ and *τ*_*p*_ would scale oppositely with respect to electron density if 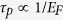
6. Similarly, for our results the scaling of *τ*_*p*_ and *τ*_*s*_ with energy suggest an EY mechanism near the Dirac point and a DP mechanism at higher energies, but Figs. 3 and 4 indicate a richer behavior. Therefore, to determine the spin relaxation mechanism it would be more appropriate to study how *τ*_*s*_ and *τ*_*s*_ scale with defect density or mobility at each value of the electron density. We stress that the decay of the spin lifetime with increasing impurity density (for the hBN substrate) is reminiscent of the conventional Elliot-Yafet mechanism, but is actually a totally different mechanism, being driven by dephasing effects in a ballistic regime.

It should be noted that our simulations are performed using a constant Rashba spin-orbit coupling, *λ*_*R*_, which is attributed to substrate effects (mirror symmetry breaking and interface interaction). In the experiments, by applying large electrostatic coupling to reach higher charge densities, an additional electric-field dependent *λ*_*R*_ should be at play. This might explain why, especially for the hBN substrate, the simulations show a larger variation of *τ*_*s*_ in energy than the gate voltage dependent spin lifetimes reported in experiments^13^,^14^.

Finally, in a recent experiment by Guimarães and coworkers, external magnetic and electric fields were used to investigate the spin lifetime anisotropy in hBN-encapsulated graphene 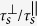 was found to range between 0.6 to 0.75 by varying the electric field. The origin of such values and their variation or possible connection to out-of-plane fields^49^ remains to be understood. Indeed, this anisotropy factor provides important information for understanding the microscopic origin of spin relaxation. In our simulations, the DP mechanism dominates for sufficiently strong disorder (such as electron-hole puddles on SiO_2_ substrates). However the case of the ultraclean hBN substrate is more complex. Here, the transport time becomes larger than the spin precession frequency, making the DP mechanism inefficient. As discussed in the Supplementary Material, for in-plane spin injection, additional effects are needed to yield spin relaxation, such as an external perpendicular magnetic field (as in Hanle spin precession measurements). More experimental and theoretical work remains to be done to fully determine the various mechanisms at play and the spin lifetime anisotropy in the limit of ultraclean graphene devices.

### Model of homogeneous SOC and electron-hole puddles

The tight-binding (TB) Hamiltonian for describing spin dynamics in graphene is given by


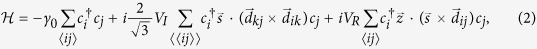


where *γ*_0_ is the nearest-neighbor *π*-orbital hopping, *V*_*I*_ is the intrinsic SOC, and *V*_*R*_ is the Rashba SOC. In the low-energy limit, this Hamiltonian is often approximated by a continuum model describing massless Dirac fermions in a single Dirac cone, 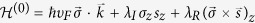, where *v*_*F*_ is the Fermi velocity, 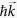 is the momentum, 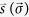 are the spin (pseudospin) Pauli matrices, 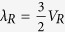, and 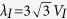. The value *λ*_*I*_ = 12 *μ* eV is commonly used for the intrinsic SOC of graphene^4^ while the Rashba SOC is electric field-dependent. Here, we let *λ*_*R*_ = 37.4 *μ*eV, taken from an extended *sp*-band TB model for graphene under an electric field of a few V/nm^4^,^5^. Higher-order SOC terms in the continuum model beyond 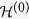 allow an extension to higher energy^50^. We note that the single cone approximation can be inappropriate in case of strong valley mixing.

### Spin dynamics methodology

The time-dependent spin polarization of propagating wavepackets is computed through^36^


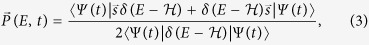


where 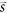 are the Pauli spin matrices and 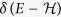 is the spectral measure operator. The wavepacket dynamics are obtained by solving the time-dependent Schrödinger equation^42^, starting from a state 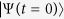 which may have either out-of-plane (*z*-direction) or in-plane spin polarization. An energy broadening *η* is introduced for expanding 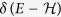 through a continued fraction expansion of the Green’s function^42^, and mimics an effective disorder. This method has been used to investigate spin relaxation in gold-decorated graphene^36^. Here, we focus on the expectation value of the spin *z*-component 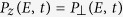 and the spin *x*-component 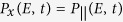.

## Additional Information

**How to cite this article**: Tuan, D. V. *et al.* Spin dynamics and relaxation in graphene dictated by electron-hole puddles. *Sci. Rep.* **6**, 21046; doi: 10.1038/srep21046 (2016).

## Supplementary Material

Supplementary Information
