# Supplementary material for: Spin dynamics and relaxation in graphene dictated by electron-hole puddles
Source: Sci Rep. 2016 Feb 15;6:21046. doi: 10.1038/srep21046 (PMC4753444; doi:10.1038/srep21046)
Supplement: Supplementary Information [file srep21046-s1.pdf]

Supplemental Material: Spin dynamics and relaxation in graphene dictated by electron-hole puddles

Dinh Van Tuan[1], Frank Ortmann,[2], Aron W. Cummings[1], David Soriano[1], & Stephan Roche,[1,3]

[1]*ICN2 - Institut Català de Nanociència i Nanotecnologia, Campus UAB, 08193 Bellaterra (Barcelona), Spain*

[2]*Institute for Materials Science, Dresden Center for Computational Materials Science, Technische Universität Dresden, 01062 Dresden, Germany*

[3]*ICREA, Institució Catalana de Recerca i Estudis Avançats, 08070 Barcelona, Spain*

In this Supplemental Material, we provide additional numerical results for the situation where spin relaxation is mainly driven by spin dephasing in absence of real space random potential fluctuations (electron-hole puddles).

We start by illustrating the scaling behavior between the numerically obtained spin relaxation time τ_s, the spin precession time T_Ω and the chosen Rashba SOC λ_R (given in the main manuscript):

$$\tau_s(E) \approx \alpha(E) T_\Omega \approx \alpha(E) \frac{\pi\hbar}{\lambda_R} \tag{1}$$

We compare the spin dynamics of the pristine graphene sample with the SOCs enhanced 100 times, i.e. $\Lambda_R = 3.74$ meV and $\Lambda_I = 1.2$ meV. The rescaled spin relaxation time for these parameters is shown in Fig.S1 (red solid line) superimposed with the case $\lambda_R = 37.4\mu$eV and $\lambda_I = 1.2\mu$eV (black solid line).

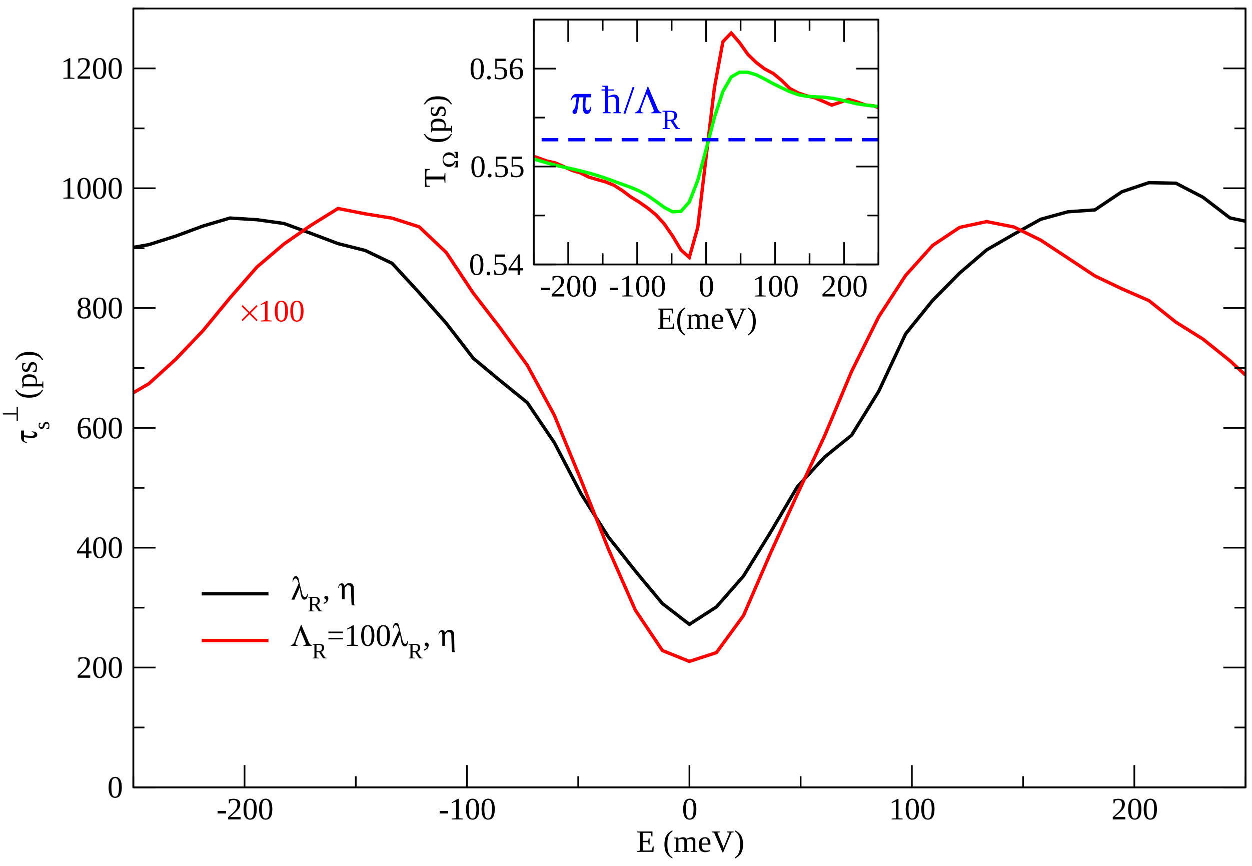

Figure 1: (Color online) Extracted spin relaxation time $\tau_s^{\perp}$ with energy broadening $\eta = 13.5$ meV (black solid line) in comparison with the scaled $\tau_s^{\perp}$ for the case of enhanced parameters ($\Lambda_R = 3.74$ meV and $\Lambda_I = 1.2$ meV). Inset: spin precession times T_{Ω} for $\eta = 13.5$ meV (red solid line) and for $2\eta = 27$ meV (green solid line) with $\Lambda_R = 3.74$ meV and $\Lambda_I = 1.2$ meV.

The energy dependence of the rescaled spin relaxation time in Fig.S1 is the almost same as with the original SOC strength. The absolute values are about 100 times smaller. The inset of Fig.S1 shows the fine structure of the spin precession time for the case of enhanced SOCs which exhibits a strong energy dependence close to the Dirac point which is driven by spin-orbit-induced bandstructure changes and which can explain the fastest spin relaxation at the Dirac point. Although the deviation of the spin precession time from $\frac{\pi\hbar}{\Lambda_R}$ is relatively small, the variation $\Delta T_\Omega/\Delta E$ is largest at the Dirac point, and increases with decreasing energy broadening of states.

By taking into account the almost perfect scaling behaviour of spin precession frequency and spin relaxation times in the considered energy interval, one obtains Eq. (1) (which is also given in the main manuscript). More specifically, this means that the spin polarization is lost during the wavepacket propagation after a few precession periods, i.e. relaxation is bound to precession which supports the dephasing nature of the spin relaxation mechanism.

In Fig.S2, we further show the spin relaxation times τ_s for in-plane and out-of-plane initital spin polarization for two values of energy broadenings $\eta = 13.5$ meV and $2\eta = 27$ meV. The spin precession times for all cases T_Ω are also shown at the same scale, but show no significant difference, with an almost energy-independent value very close to $T_\Omega^{||} = T_\Omega^{\perp} = \frac{\pi\hbar}{\lambda_R} \approx 55$ (ps) which is in perfect agreement with average value given by analytical considerations [1]. The extracted spin relaxation times however show some significant energy dependence with the energy broadening, with a approximate $\tau_s \sim 1/\eta$ scaling behavior. The M-shape of spin relaxation time with a minimum close to Dirac point (which is related to the spin-pseudospin locking already mentioned in

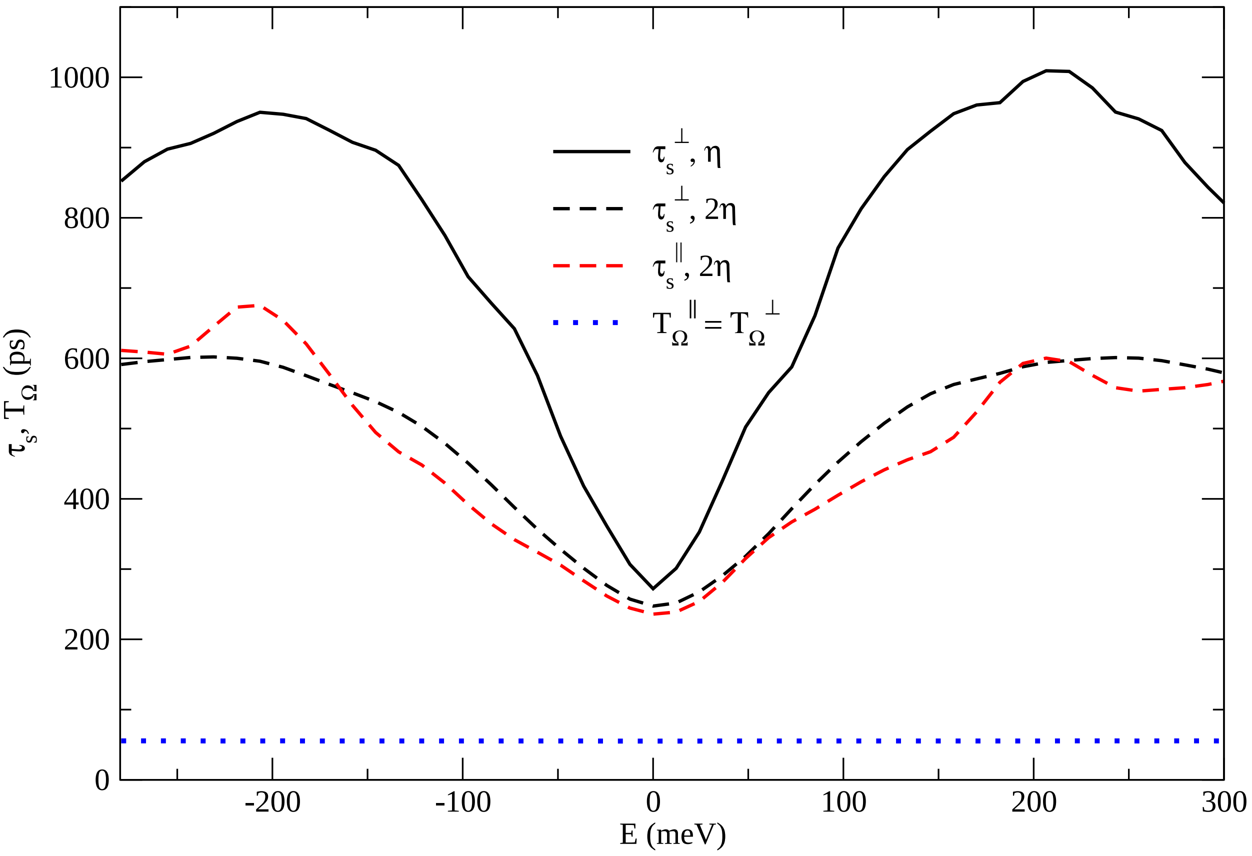

Figure 2: (Color online) Extracted out-of-plane spin relaxation time $\tau_s^{\perp}$ with two energy broadenings $\eta = 13.5$ meV (black solid line), $2\eta = 27$ meV (black dashed line) and the spin relaxation time $\tau_s^{||}$ (red dashed line) of the precessing component of the in-plane case with energy broadenings 2η. The spin precession periods T_Ω in both cases are shown in the dotted line.

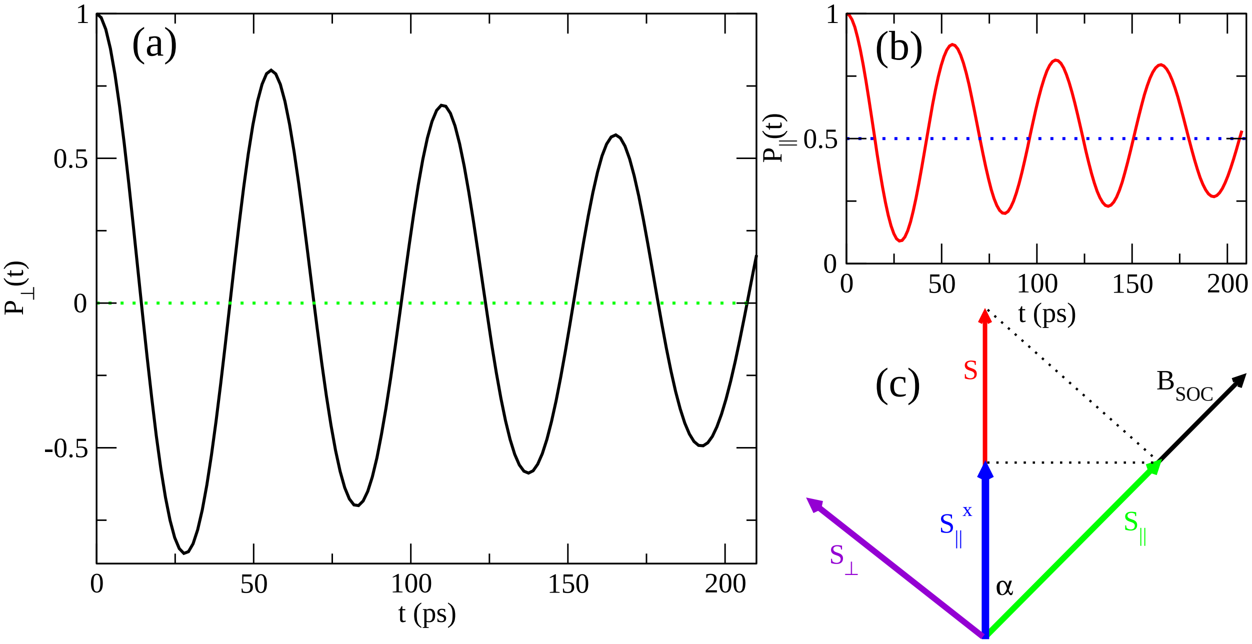

Figure 3: (Color online) Spin polarizations for out-of-plane (a) and in-plane (b) spin injections. (c): Illustration of parallel (green) and perpendicular (violet) components. The projection of parallel component in x direction is shown in blue.

Ref.[2]) has also been found for the hBN puddle case in the main manuscript. A similar M-shape but related to another physical mechanism for spin relaxation has been discussed for SiO_2 puddles in the main text.

One notices in Fig.S2 that the extracted spin relaxation time $\tau_s^{||}$ of the precessing component in the in-plane case (red dashed line) is almost identical to out-of-plane spin relaxation time $\tau_s^{\perp}$ (black dashed line) indicating that both are governed by the same relaxation mechanism. It is also worth noting that the parallel-spin components have a more rich structure of spin dynamics because the Rashba spin-orbit interaction creates in-plane effective magnetic fields.

This effect is further discussed in Fig. S3 where the time-evolution of spin polarization with initial out-of-plane (Fig.S3(a)) and in-plane (Fig.S3(b)) spin injection are reported. Both spin signals follow $cosine$ oscillation functions with the same period T_Ω, but differently to the out-of-plane initital polarization (which oscillates around zero and is thus eventually lost at long enough elapsed times), the in-plane initial polarized spin keeps a finite polarization in the long time limit of 1/2 the initial value. This effect is rationalized as follows. Fig.S3 (b) shows that the spin polarization of the in-plane spin injection oscillates around 0.5 and is well fitted by the function

$$P_x(t) = \frac{1}{2} + \frac{1}{2} cos\left(\frac{2\pi t}{T_\Omega}\right) e^{-t/\tau_s^{||}} \tag{2}$$

whereas the out-of-plane one oscillates around 0 (Fig.S1 (a)) and is well fitted by

$$P_z(t) = cos\left(\frac{2\pi t}{T_\Omega}\right) e^{-t/\tau_s^{\perp}} \tag{3}$$

In both fitting forms, the relaxation term e^{-t/τ_s} is always bound to the precession term $cos\left(\frac{2\pi t}{T_\Omega}\right)$. The time-independent term in Eq.(2) can be understood from the direction of the magnetic field $\mathbf{B}_{SOC}$ induced by SOCs.

Indeed, an arbitrary spin $\mathbf{S}$ precesses about $\mathbf{B}_{SOC}$, and such precessing spin can be considered as the combination of two components $\mathbf{S} = \mathbf{S}_{||} + \mathbf{S}_{\perp}$ (see Fig.S1(c)). The parallel component $S_{||} = S\cos\alpha$ (with α being the angle between spin and magnetic field direction and varing from 0 to π depending on the direction of motion) points along the magnetic field direction and does not precess while the perpendicular component $S_{\perp} = S\sin\alpha$ rotates about the magnetic field with the

period T_Ω, corresponding to the factor $cos\left(\frac{2\pi t}{T_\Omega}\right)$ in Eq. (2). Because electrons can move in arbitrary directions, α varies from 0 to π. Accordingly by averaging the projections of the parallel-spin components in the x direction (the initially injected spin direction) gives

$$\overline{S^x_{||}} = \overline{S_{||}\cos\alpha} = S\overline{\cos^2\alpha} = \frac{1}{2}S \quad (4)$$

This corresponds to the first term in the right hand side of Eq. (2). The factor $\frac{1}{2}$ in the time dependent term of Eq. (2) can be also obtained in the same manner. While the parallel-spin component (the first term in Eq. (2)) does not relax, the rotating term is always followed by an exponential decay, again establishing that spin precession induces spin dephasing as a dominant effect on spin relaxation. In the literature [1,3], the magnetic field induced by SOC is in plane. This leads to the disappearance of the parallel component in Eq.(3) because spin is initially injected out-of-plane. The only remaining term related to the precessing component also involes the relaxation.

We finally note that such robust spin polarization for in-plane injection is further relaxed by additional perpendicular fields, such as those applied in non-local Hanle spin precession measurements [4].

1 References

1. Ertler, C., Konschuh, S., Gmitra, M. & Fabian, J. Electron spin relaxation in graphene: The role of the substrate. *Phys. Rev. B* **80**, 041405 (2009).

2. Van Tuan, D., Ortmann, F., Soriano, D., Valenzuela, S. & Roche, S. Pseudospin-driven spin relaxation mechanism in graphene. *Nature Physics* **10**, 857 (2014).

3. Zhang, P. & Wu, M. Electron spin diffusion and transport in graphene. *Phys. Rev. B* **84**, 045304 (2011).

4. Roche, S. & Valenzuela, S. O. Graphene spintronics: puzzling controversies and challenges for spin manipulation. *Journal of Physics D: Applied Physics* **47**, 094011 (2014).
